# Investigation of the Anti-Inflammatory and Anti-Oxidant Activities of a Novel Kaempferol-Liposome-Loaded Hydrogel for the Treatment of Acute Eczema

**DOI:** 10.3390/gels11020083

**Published:** 2025-01-22

**Authors:** Yuqing Fang, Yong Liu, Keang Cao, Wang Shen, Lu Wang, Bin Sun, Yongli Zhang, Qin Zhang, Hongmei Xia

**Affiliations:** College of Pharmacy, Anhui University of Chinese Medicine, Hefei 230012, China; 2023205221005@stu.ahtcm.edu.cn (Y.F.); 13034049882@163.com (Y.L.); 2023205221003@stu.ahtcm.edu.cn (K.C.); 15155034624@163.com (W.S.); 15155063158@163.com (L.W.); 13721231091@163.com (B.S.); 18119742560@163.com (Y.Z.); 2020205221001@stu.ahtcm.edu.cn (Q.Z.)

**Keywords:** kaempferol, liposome-loaded hydrogel, acute eczema

## Abstract

Kaempferol liposome hydrogel is a novel drug carrier designed to solve the problems of the poor water solubility and low bioavailability of kaempferol. By combining kaempferol with liposomes and further forming a hydrogel, this composite formulation not only improves the stability of the drug but also enhances its penetration and therapeutic effect on the skin. It was demonstrated that the liposomal hydrogel of kaempferol had ex vivo and in vivo antioxidant activities, which could effectively inhibit the inflammatory response and oxidative stress and, thus, showed significant efficacy in the treatment of acute eczema. In the acute eczema model, kaempferol liposome hydrogel significantly improved the skin condition of mice by reducing the symptoms of skin redness, swelling, and itching. The experimental results showed that the hydrogel was rapidly absorbed into the skin after application and continued to release the drug to maintain its efficacy for a longer period of time. In addition, the kaempferol liposome hydrogel also showed good physicochemical stability and was not easily discolored or separated, making it suitable for long-term use. As an innovative drug carrier for the treatment of acute eczema, the kaempferol liposome hydrogel shows good application prospects and provides a new treatment option for eczema patients.

## 1. Introduction

Acute eczema (AE) is an inflammatory skin disease. There are some clinical features, including dryness, itching, and eczema in the skin, accompanied by increased transcutaneous water loss [1,2]. AE involves cytokines expressed in T-lymphocytes, T-helper cell type 1 (Th1) cells, T-helper cell type 2 (Th2) cells, and dendritic cells. Th1 cells secrete interleukin (IL)-2, interferon (IFN), and tumor necrosis factor (TNF) to activate macrophages, while Th2 cells secrete cytokines, such as IL-4, IL-5, IL-6, IL-10, and IL-13, to increase the production of immunoglobulin E (IgE) and induce mast cells and eosinophils to differentiate [3,4]. Th2 cytokines directly affect skin cells. At present, the drugs for treating AE mainly include corticosteroids, antihistamines, and antibiotics. However, they have side effects during long-term use, such as skin atrophy and telangiectasia [5]. Therefore, people have been trying to find safer and more effective plant-derived compounds that can regulate the pathological mechanism of AE.

Kaempferol (KAE) has been reported to inhibit the release of inflammatory cytokines. For example, KAE reduces the expressions of inflammatory factors by inhibiting the NF-κB signaling pathway [6,7]. In addition, it inhibits oxidative stress, reduces free-radical damage to the skin, and modulates the immune response by inhibiting T-lymphocyte production and activation, thereby reducing immune-mediated inflammation [8]. KAE can also reduce the intrusion of external irritants and allergens by promoting the expressions of skin barrier proteins, such as keratin, and works through various signaling pathways, including MAPK and other pathways [9]. KAE exerts its therapeutic effects on eczema through a variety of mechanisms, including anti-inflammatory, antioxidant, modulation of the immune response, and improvement of the skin barrier function. These mechanisms of action make KAE a potentially effective ingredient for the treatment of eczema [10].

Liposomes (LPs) are a kind of nanocarrier capable of encapsulating drugs, with a good encapsulation ability for hydrophilic and hydrophobic drugs. LPs protect drug molecules from enzymatic degradation in vivo or from the external environment through a bilayer membrane structure, thus improving drug stability [11,12,13]. However, LPs alone suffer from the problem of hasty or uneven release, resulting in low drug utilization. On the other hand, hydrogels are highly absorbent materials with a three-dimensional network structure, which can provide good, slow drug release. The hydrogel can distribute the drug uniformly within it and prolong the action time of the drug by controlling the release rate of the drug [14,15]. However, hydrogels (GELs) themselves have limitations in terms of drug stability, such as degradation or dissolution under certain conditions. The combination of the two can give full play to their respective advantages, and in the sorbitol liposome hydrogel, as a novel form of drug carrier, sorbitol is difficult to dissolve in water and is metabolized faster in vivo, and its range of action is not concentrated, which can easily lead to enhanced side effects. By combining sorbitol with LPs and further encapsulating it in a hydrogel, the stability of the sorbitol can be significantly improved, and the rapid release of the drug and its wastage can be reduced, thereby improving the bioavailability of KAE. When LPs are combined with hydrogels, this composite system not only achieves the slow release of the drug but also further controls the release rate of the drug through the network structure of the hydrogel, thus enabling more precise targeted drug delivery [16,17]. This form of KAE not only better exerts pharmacological effects, such as anti-inflammatory and antioxidant effects, but also acts more effectively on the skin through controlled release, thus improving the therapeutic effect. In contrast, most flavonoids (e.g., edible ferns [18]) are usually applied to eczema treatment in the free form or in simple preparations, but they are less bioavailable and prone to first-pass metabolism, resulting in lower effective concentrations in the body. As a result, they usually need to be taken orally, and their effects may not be as drastic as those of KAE liposomal hydrogels.

## 2. Results and Discussion

### 2.1. Network Pharmacology Results

**Figure 1:**
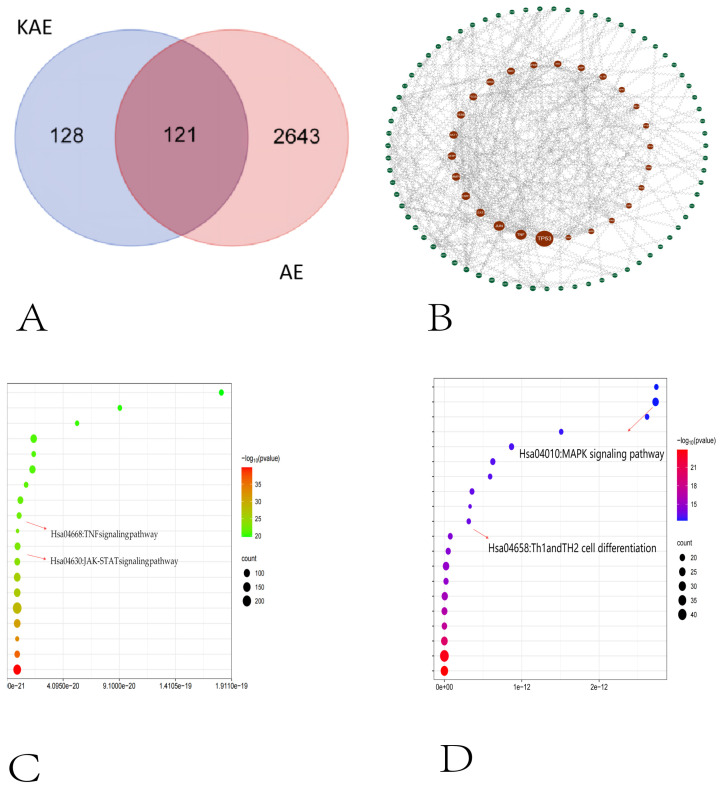
Network pharmacology analysis. (**A**) Venn plot of the target of the action of KAE with AE. (**B**) PPI network of drug–disease targets for KAE treatment of AE. (**C**) Bubble plot for the signal analysis of the GO enrichment analysis of potential targets for KAE treatment of AE. (**D**) Bubble plot for the signal analysis of the KEGG enrichment analysis of potential targets for KAE treatment of AE.

The results of the GO functional enrichment analysis showed that the cytokine–cytokine receptor interaction, TNF signaling pathway, JAK-STAT signaling pathway, etc., were involved in the process of the inflammatory response and reduced the occurrence and development of inflammation (Figure 1C); the results of the KEGG functional enrichment analysis showed that the Th1 and Th2 cell differentiation, MAPK signaling pathway, etc. were also related to inflammation, as shown in Figure 1D.

### 2.2. Molecular Docking Results

**Figure 2:**
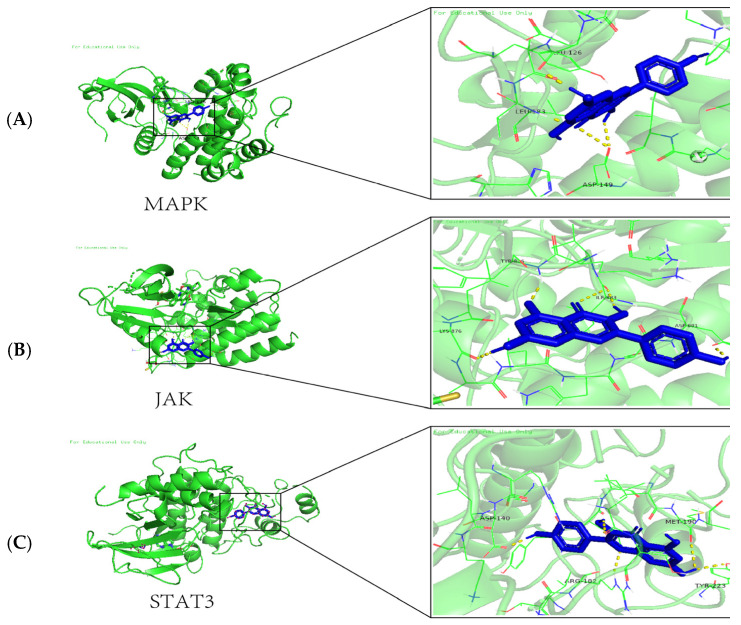
Results of KAE docking with key targets, including (**A**) MAPK, (**B**) JAK, and (**C**) STAT3.

The top three core components are molecularly docked with key targets. It is generally assumed that the lower the energy of the conformational stability of the ligand–receptor binding, the greater the likelihood of an action occurring. The docking results showed that the binding energies of the selected proteins and the corresponding small molecules were <−6.3 kJ/mol, and the binding energies of KAE to MAPK, JAK, and STAT3 were −6.3 kJ/mol, −6.8 kJ/mol, and −7.01 kJ/mol, respectively, indicating that the compounds and the receptor had a good binding activity, as shown in Figure 2.

### 2.3. Cumulative Release Rate of KAE from Its Formulations In Vitro

**Figure 3:**
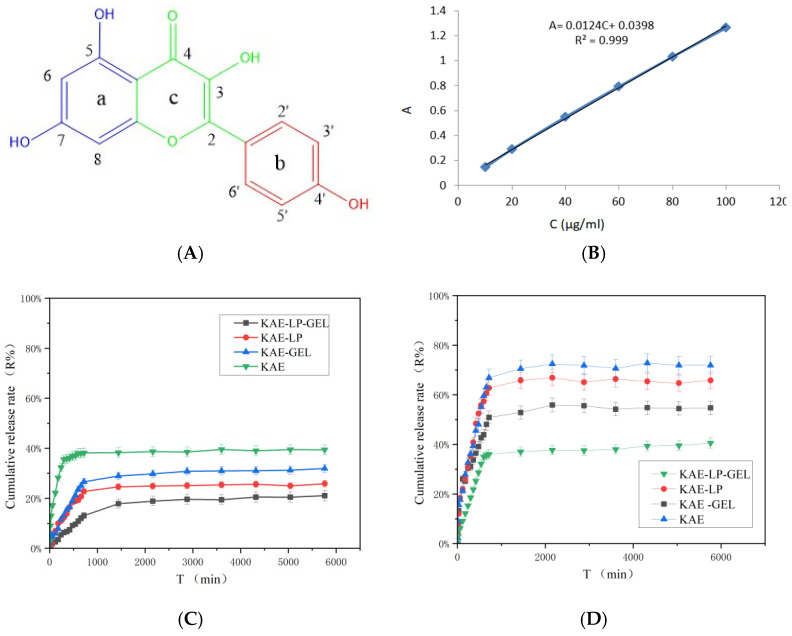
Physicochemical properties and in vitro release characteristics of KAE and its preparations. (**A**) The structural formula of KAE; (**B**) the standard curve of KAE; (**C**) cumulative release rate of KAE and its preparations across the isolated skin of mice ex vivo; (**D**)cumulative release rate of KAE and its preparations across the dialysis membrane in vitro.

The molecular formula of KAE is C_15_H_10_O_6_, as shown in Figure 3A, which belongs to flavonoids, and its core structure contains multiple phenolic hydroxyl groups (i.e., -OH groups), and these phenolic hydroxyl groups are one of the important moieties for KAE to exert its medicinal effects. In addition, KAE has a diphenylpropane structure, which is also an important structural basis for its medicinal effects. The standard curve was plotted using absorbance (A) as the vertical coordinate and the concentration (C) of KAE as the horizontal coordinate. As shown in Figure 3B, there was a good linear relationship between the KAE concentration and absorbance in the range of 0.01–0.1 mg/mL. In the in vitro release experiments, the cumulative permeability curves of KAE and its formulations were plotted using the cumulative permeability of the drug, “R%”, as the vertical coordinate and the time, “T”, as the horizontal coordinate (Figure 3C,D). During the experiment, the cumulative release of the KAE was the highest, and that of the KAE-LP-GEL was the lowest. This might be due to the fact that free KAE is not restrained by the LPs and hydrogel during drug release. KAE-LP-GEL combined the dual retardation mechanism of the LPs and hydrogel, in which the drug was first encapsulated in LPs and then embedded in the network structure of the hydrogel. This dual carrier structure further prolonged the release time of the drug, resulting in a relatively low cumulative release rate.

### 2.4. Characteristics of the KAE-Loaded LPs, KAE-Loaded Hydrogel, and KAE-LP-Loaded Hydrogel

**Figure 4:**
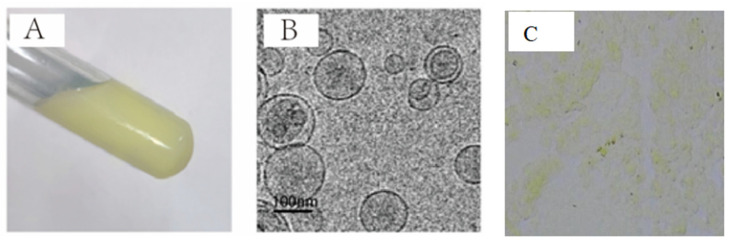
Morphology of KAE-LP: (**A**) exterior appearance; (**B**) cryo-EM diagram; (**C**) optical micrograph.

In the preparation process of the LPs, the encapsulation efficiency was better when the mass ratio of the phospholipid to the cholesterol was 3:1. According to the calculation result of Equation (1), the encapsulation efficiency, particle size, zeta-potential, and polydispersity index of KAE-LP were 84.33 ± 6.02%,161.2 ± 18.5 nm, −37.2 ± 4.6 mV, and 0.215 ± 0.031, as shown in Figure 4A,B; 1% CBP 934 was used as the hydrogel matrix, as shown in Figure 4C. The viscosity of the KAE-loaded hydrogel was moderate, and the fluidity was good; the whole system was relatively uniform and stable, and it was a colorless, tasteless, and transparent hydrogel. The KAE-LP-loaded hydrogel was in a translucent state. The characteristics of these preparations are shown in Table 1.

### 2.5. Bodyweight, Skin Thickness, and Antioxidant Activities of KAE and Its Preparations in Mice

**Figure 5:**
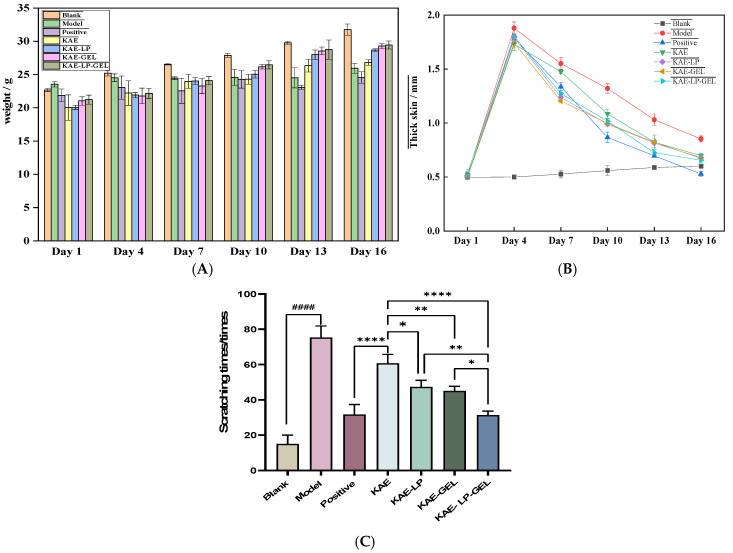
Physical indicators and behaviors of mice. (**A**) Effects of DNCB-induced changes on bodyweight in mice; (**B**) effects of DNCB-induced changes on skin thickness in mice; (**C**) scratching times in each group of mice in 20 min. Comparison of the blank and model groups, #### *p* < 0.0001; comparisons of the KAE group with the KAE-LP group, KAE-GEL group, and KAE-LP-GEL group, * *p* < 0.05, ** *p* <0.01, and **** *p* < 0.0001.

The changes in the bodyweight of the mice during 16 days are shown in Figure 5A. The bodyweight of the mice in the blank group was increasing all the time, indicating that the mice in the blank group were in good physical condition. In the first four days, the bodyweight of the mice in the model group increased only a little because of the stimulation of DNCB, and from day 4 until the end, the mice in the model group basically did not increase in bodyweight. The mice in the positive control group also gained some weight first, then lost weight again because of the eczema model, and then gained weight again because the skin inflammation had been alleviated, but because of the side effects of the compound dexamethasone acetate cream on the skin, the mice in the positive control group became very thin and small, and the final weight was the lightest, and the mice in each drug treatment group kept increasing their weight slowly. Overall, the bodyweight of the mice in the model group was lower compared with that of the mice in the blank group; the bodyweight of the mice in each drug-treated group was heavier compared with that of the mice in the model group, and the bodyweights of the mice in the KAE-LP, KAE-GEL, and KAE-LP-GEL groups were heavier than that of the mice in the KAE group, indicating that KAE-LP, KAE-GEL, and KAE-LP-GEL dosage forms were more effective than free KAE in the treatment of AE mice. The bodyweights of the mice in the KAE-GEL and KAE-LP-GEL groups were heavier than those of the mice in the other groups, which might be because of the hydrogel adhering more to the skin. The changes in the skin thickness on the backs of the mice over 16 days are shown in Figure 5B. On the first day of the experiment, the skin thickness in the seven groups was almost the same. Under DNCB sensitization, the skin thickness of the mice thickened a lot on the fourth day, except for the blank group, where the skin grew thicker naturally because of the increase in the bodyweight. The skin thickness in the other six groups of mice generally increased first and then decreased. The skin thickness of the mice in the model group was higher than that of the mice in the blank group, and the skin thickness of the mice in the positive control group and each drug-treated group with treated eczema skin inflammation was lower than that of the mice in the model group. The treatment effect in the KAE-LP-GEL group was better than those in the KAE-LP and KAE-GEL groups, and the performances in the preparation groups (KAE-LP, KAE-GEL, and KAE-LP-GEL) were better than that in the active-pharmaceutical-substance (KAE) group.

### 2.6. Test Results of Scratching Times for KAE and Its Preparations in Mice

Under normal circumstances, the mice in the blank group had fewer scratching and fidgeting behaviors, and there was no obvious abnormal behavior. In the AE model induced by DNCB, the mice scratched more frequently, and there were more fidgety behaviors, such as licking claws and heads. The scratching behaviors of the mice in each group were observed within 20 min after the last administration, and the experimental results are shown in Figure 4C. The scratching frequency of the mice in the model group was significantly increased compared to that of the mice in the blank group; the scratching frequencies were decreased in the preparation groups (KAE-LP, KAE-GEL, and KAE-LP-GEL) compared to that of the mice in the KAE group.

### 2.7. AE Modeling Results and Skin Inflammation Score Test Results

**Figure 6:**
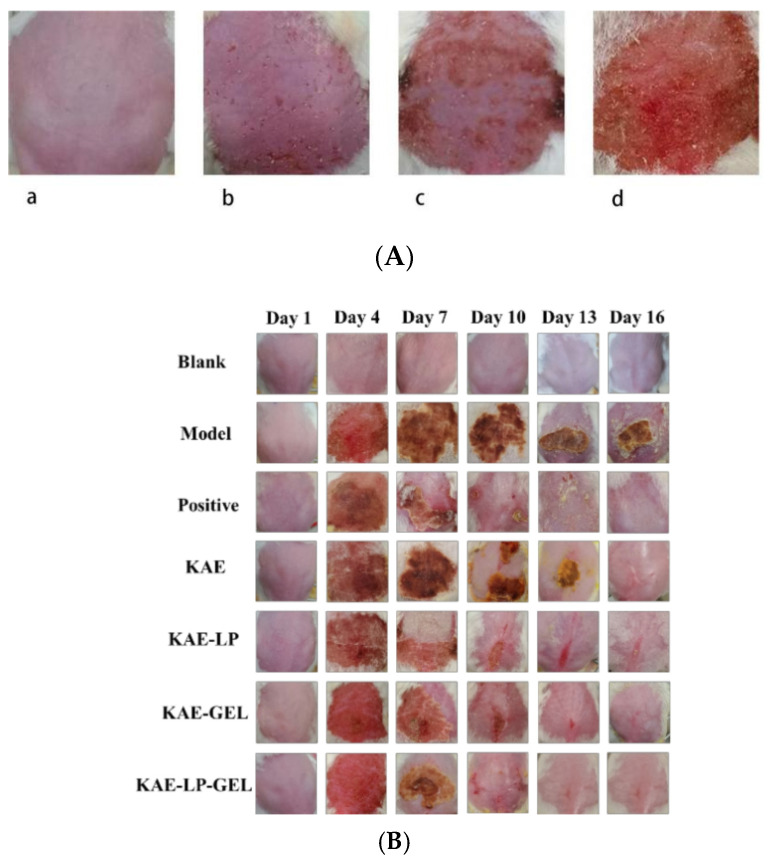

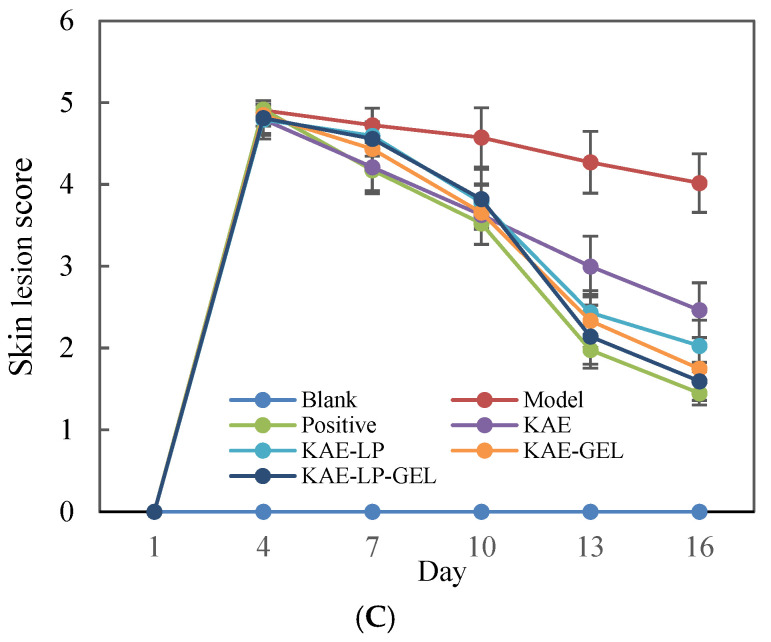
Modeling of AE in mice, showing different courses of drug administration and the scoring of lesions. (**A**) The process of dorsal skin injury in a mouse model of AE; (**B**) the course of the skin lesions in each group of mice from day 1 to day 16 after administration of DNCB; (**C**) the scoring of the dorsal skin lesions in each group of mice from day 1 to day 16 after administration of DNCB.

Acute eczema-like skin damage could be induced by repeatedly exposing the dorsal skin and ears of the mice to DNCB. As shown in Figure 6B, the back skin of the mice in the model group had skin characteristics, such as redness, papules, exudation, and scabbing, which reached the peak on the fourth day. Except for the blank group, the back skin of the mice was seriously damaged, and the dermatosis score was about 6. In the model group, the skin on the backs of the mice was dry and desquamated, and the epidermal barrier was destroyed, resulting in severe scabbing. However, the degree of skin damage was gradually reduced through the self-healing ability of the mice, but there was still severe redness and scabbing. With the treatment, the skin inflammation of the mice was alleviated, the damage of the epidermal barrier was repaired, and erythema and edema were gradually eliminated. From the first day of the DNCB-induced mouse skin, the skin was scored; see Figure 6C for the scores of the skin lesions on the backs of the mice within 16 days. The results showed that the scores of the skin lesions in the model group were higher than those of the skin lesions in the blank group and that the scores of the skin lesions in the drug treatment group were lower than those of the skin lesions in the model group. By observing the changes in the skin appearance and the results of the skin lesion scores in the mice, it was shown that KAE and its preparations could alleviate AE-like symptoms in mice, among which the LP-loaded hydrogel had the most obvious therapeutic effect on the AE.

The normal mice had smooth fur, were responsive, and had bright hair, and no abnormal behavior occurred. The skin on the backs of the mice was dehaired in the afternoon of the day before the experiment, and the skin in the dehaired area was undamaged, smooth, and red (Figure 6A(a)) On the first day, 5% DNCB was applied to sensitize the mice, and on the second day, the skin on the backs of the mice showed mild erythema and edema (Figure 6A(b)), and the mice began to scratch and showed irritability and jumping and other behaviors. On the third day, the sensitization was followed, and the skin on the backs of the mice began to ooze and crust (Figure 6A(c)). On the fourth day, the back skin of the mice in the model, positive, KAE, KAE-LP, KAE-GEL, and KAE-LP-GEL groups showed very serious edema, redness, bulging, crusting, and other typical AE skin characteristics (Figure 6A(d)), proving that the modeling was successful; at this time, the mice’s scratching behavior was more serious, and their responses were more sluggish.

### 2.8. The Results of the Ear-Swelling Measurements

Photographs of the left and right ears of the mice were taken before weighing, and the experimental results of the ear inflammation and swelling in each group of mice are shown in Table 2. Except for the blank group, there were obvious differences in the health of the left and right ears of each mouse in the other groups. The left ear was red, swollen, and congested because of the stimulation of 0.5% DNCB. The left ear was much larger than the right ear, and the left ear was also heavier than the right ear. Compared with the mice in the blank group, the ear-swelling degree in the model group was 0.0041 ± 0.0001 g, and the swelling inhibition rate was 59.16 ± 5.69%, both of which were significantly increased. The ear-swelling degrees and swelling inhibition rates of the mice in the positive control group and drug administration group were also higher than those in the blank group. Compared with the model group, the inflammation and swelling of the mouse ears in each drug treatment group were significantly inhibited, and the swelling degree and swelling inhibition rate were significantly reduced.

### 2.9. Measurement Results of Spleen and Thymus Indices

**Figure 7:**
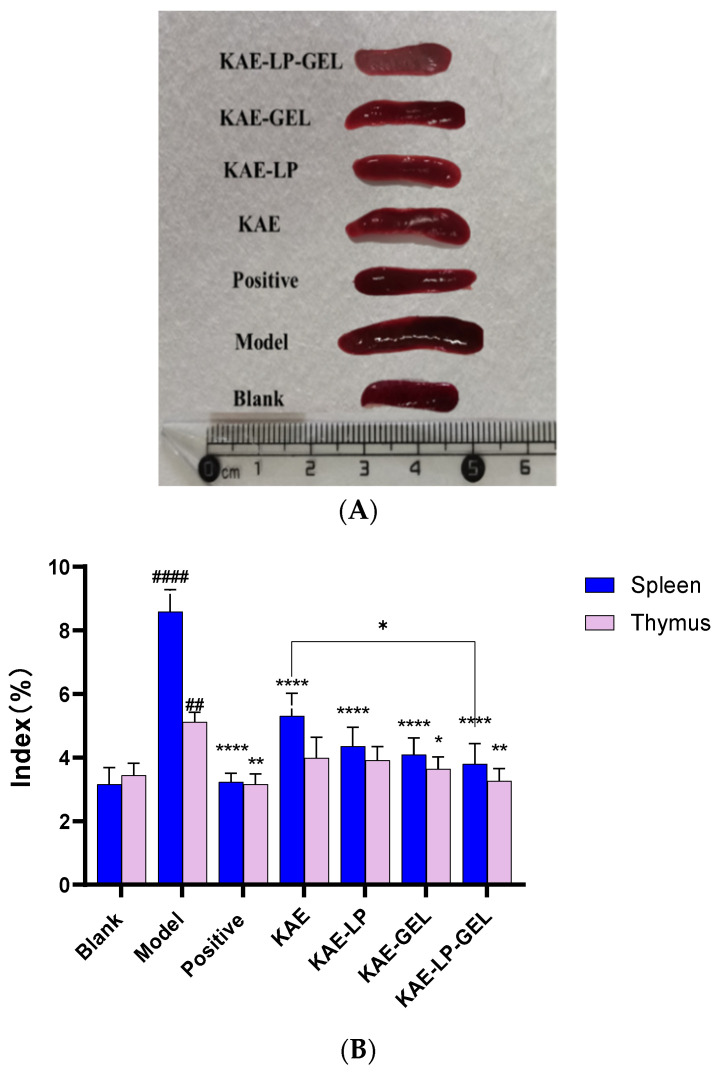

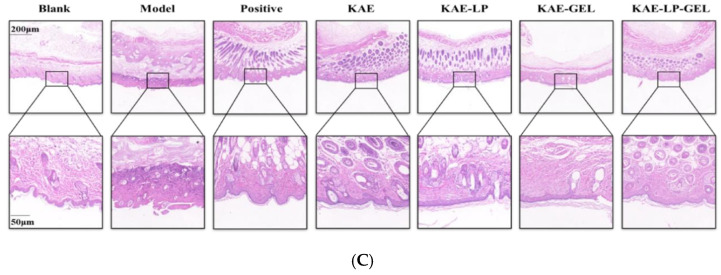
Effect of DNCB induction on the spleens and thymuses of the mice. (**A**) Size of the spleen in each group of mice; (**B**) Spleen and thymus index of mice in each group (**C**) The skin injuries of the mice in each group were observed under a microscope after HE staining.

As shown in Figure 7A, compared with the blank group mice, the sizes of the spleen and thymus of the mice in the DNCB-induced AE model group increased significantly, but they decreased in the drug-treated groups after treatment. As can be seen from Figure 7B, compared with the blank group, the indices of the spleen and thymus in the model group increased. The indices of the spleen and thymus in the positive control group and the KAE-LP, KAE-GEL, and KAE-LP-GEL groups decreased significantly after the treatment and were lower than those in the model group. Spleen thymus of mice in the model group compared to the blank group was #### *p* < 0.0001 and ## *p* < 0.01, respectively; spleen of mice in the treatment group compared to the model group was **** *p* < 0.0001. Thymus of mice in the Positive group, the KAE-GEL group, and the KAE-LP-GEL group compared to mice in the model group was ** *p* < 0.01; ** *p* < 0.01; * *p* < 0.05; spleen in KAE group and KAE-LP-GEL group compared to * *p* < 0.05.

### 2.10. The Results of the Histopathological Sections

The skin damage in each group of mice was observed under the microscope after HE staining, and the results are shown in Figure 7C. It could be observed that the back skin of the blank group mice was intact without any damage, while the model group mice had a thickened epidermis, obvious inflammatory cell infiltration, edema between cells, and serious skin damage. With the treatment, this proliferation was inhibited. Compared with the model group, the skin edema and inflammatory cell infiltration in the positive control group and the KAE-LP, KAE-GEL, and KAE-LP-GEL treatment groups were less severe, the KAE-LP-GEL group had an obvious therapeutic effect, and the skin was obviously thinner. Histopathological analysis confirmed that KAE-LP-GEL reduced the epidermal thickening induced by DNCB, thus alleviating the skin symptoms of AE induced by DNCB in mice.

### 2.11. The Results of the MDA Contents in the Skin and Tissues Ex Vitro

**Figure 8:**
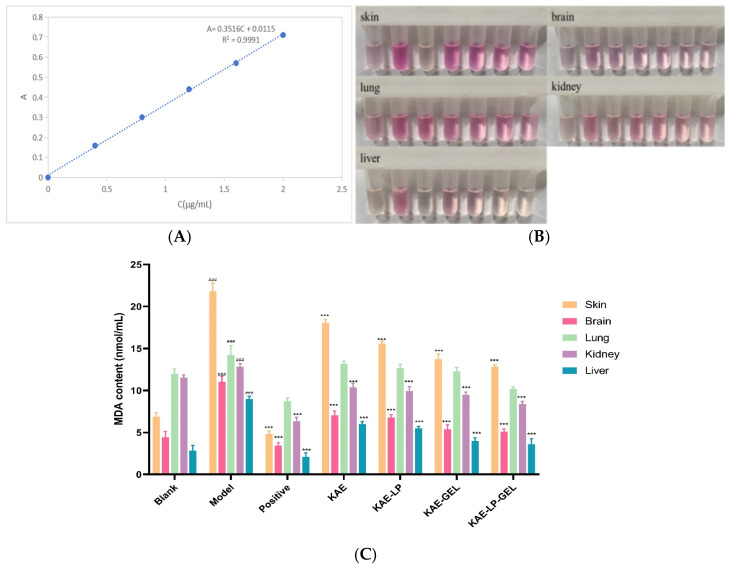
KAE-LP, KAE-GEL, and KAE-LP-GEL all exhibited potent antioxidant inhibition effects of DNCB induction on the MDA content in various tissues of mice. (**A**) MDA standard curve; (**B**) color development of the homogenates of the back skin and tissues from each group of mice; (**C**) MDA contents of skin and tissues from the mice in the different treatment groups compared with those of skin and tissues from the mice in the blank group, ### *p* < 0.001, and compared with those of skin and tissues from the mice in the model group, *** *p* < 0.001.

The final fitting-curve equation of the MDA standard curve is A = 0.3516C + 0.0115, R^2^ = 0.9991, as shown in Figure 8A. The experimental results show that MDA has a good linear relationship in the range of 0.4–3 μg/mL. According to the MDA standard curve, the content of MDA in each organ was evaluated.

Using TBA (thiobarbituric acid) assays, we explored the mechanism of the effect of AE on the contents of lipid peroxidation products in the dorsal skin and its internal tissues in mice and further evaluated the changes in these key biochemical indices in the model mice after the interventions of KAE and its preparations. As shown in Figure 8B, the shade of the color of the samples reflected the content of the MDA in the samples, with darker colors indicating higher MDA contents, reflecting higher levels of oxidative stress, and on the contrary, lighter red colors implied lower MDA contents, reflecting better antioxidant statuses and lower levels of oxidative stress. In the color development experiments of the tissue homogenates, the color development results of the mouse samples from each group clearly showed a color gradient from the blank control group to the different groups (model, positive control, KAE-LP, KAE-GEL, and KAE-LP-GEL), in which the skin tissue homogenates from the model group showed the most obviously red color, which directly indicated that the content of malondialdehyde (MDA) in the skin tissues of the mice in this group had reached the highest level. This reflected the increased lipid peroxidation reaction. The data in Figure 8C further revealed that in the mice in the positive, KAE, KAE-LP, KAE-GEL, and KAE-LP-GEL groups, the skin tissue was the main site of the lipid peroxidation product accumulation and maintained significantly higher levels than lung, kidney, and brain tissues, while the liver tissue kept relatively lower levels. Significant oxidative stress was determined in the dorsal skin and all the organs from the model group when compared with those from the blank group mice. It manifested as a sharp increase in the MDA content from 7.13 ± 0.06 nmol/mL in the blank group to 21.67 ± 0.14 nmol/mL in the model group, which highlighted the degree of oxidative damage in the pathological process of AE. It is worth noting that when KAE-LP, KAE-GEL, and KAE-LP-GEL all exhibited potent antioxidant inhibition effects on MDA production, they effectively reduced the degree of lipid peroxidation in the tissues. In particular, KAE-LP-GEL performed the best in inhibiting MDA production, outperforming LPs or gels alone, providing strong evidence for further exploration of the potential of the LP-loaded hydrogel as a novel drug delivery system in the treatment of AE and its associated oxidative-stress-induced damage.

In the DNCB-induced mouse model of AE, it was found that KAE-LP-GEL exerted a significant inhibitory effect on AE. The results of the network pharmacology analysis showed that KAE might exert its efficacy in treating AE through multi-target pathways. Studies have shown that the JAK-STAT pathway plays a key role in cytokine signaling, including cytokines, such as IL-13 and IL-4, which play important roles in the inflammatory response in AE [19,20]. The MAPK pathway is also an important target for drug action in AE because it is widely involved in inflammatory responses, immune regulation, and cell proliferation processes [21,22,23]. According to molecular docking analysis, the binding energies of KAE to the core targets of STAT, JAK, and MAPK were all less than −5.0 kcal/mol, indicating that the binding of these targets to KAE is well stabilized. The DNCB-induced mouse model exhibited epidermal thickening, marked inflammatory cell infiltration, intercellular edema, and severe skin damage, which were highly similar to the main pathological features of human AE. The characteristics are highly similar to those of human AE. These data suggest that KAE-LP-GEL attenuates the pathological changes in AE-like skin inflammation, including dermatitis scores, malondialdehyde (MDA) levels, epidermal thickening, and inflammatory cell infiltration. As a flavonoid, KAE possesses a hydroxyl group that is the key to its antioxidant action and effectively reduces oxidative stress in cells through oxygenation or scavenging of free radicals. In particular, the hydroxyl group located at the 4’ position on the B-ring synergistically enhances the free-radical scavenging ability of KAE, with the hydroxyl groups located at the 3, 5, and 7 positions on the ring, as shown in Figure 4A [24]. In addition, the hydroxyl group in KAE can exert anti-inflammatory effects by modulating signaling pathways and reducing the expressions of pro-inflammatory factors [25,26]. Transdermal and dialysis experiments showed that when KAE was encapsulated by LPs and distributed in hydrogels, the solubility of the KAE was significantly increased, enhancing its bioactivity and bioavailability. At the same time, this encapsulation achieved a continuous and stable release of the drug and reduced the fluctuations in the drug concentration, thus improving the overall therapeutic effect.

In conclusion, KAE-loaded hydrogel LPs have significant therapeutic efficacy in the treatment of AE, showing good pharmacodynamic properties. Experiments in a mouse model of AE have demonstrated the significant efficacy of the KAE liposomal hydrogel in wound healing. Existing commercial wound dressings, such as gauze and transparent films, mainly provide protection and limited exudate absorption [27] but lack the antioxidant and anti-inflammatory properties of the KAE liposomal hydrogel, and the KAE liposomal hydrogel has good air permeability and hygroscopicity, which maintain a moist environment around the wound and promote cell growth and division. Commercial dressings, such as polyurethane and hydroxyethyl cellulose membranes, also have a certain degree of breathability and hygroscopicity, but the specific performance may vary from product to product and may not necessarily have the excellent properties of the KAE liposome hydrogel [28,29]. Overall cost-effectiveness Despite the higher raw material and production costs of the KAE liposome hydrogel, its excellent wound-healing effect and comfort may lead to higher overall cost-effectiveness by reducing the consumption of healthcare resources and time during the patient’s recovery. The KAE liposome hydrogel does not have a deleterious effect on the environment during use, and its degradability may be superior to those of some existing commercial dressings, such as plastic films, which may cause long-term pollution to the environment after use. However, a comprehensive comparison with existing commercial products still requires further research. This study provides a theoretical basis for the in-depth development and clinical application of drug-loaded liposomal hydrogels.

## 3. Conclusions

The experimental results showed that the liposomal gel of the KAE not only has good antioxidant effects in vitro and in vivo but also has therapeutic effects on AE with some anti-inflammatory effects, and all the therapeutic effects of the liposomal gel are stronger than those of the APIs, which better utilize the pharmacological activity of the KAE. Oxidation and inflammation play key roles in many diseases, and it can be inferred that KAE has good potential in other diseases caused by excessive oxidation and inflammation and has great potential in the future treatment of inflammatory skin diseases. However, KAE is a refractory drug, and although the solubility and stability of the KAE can be improved by liposome technology, there are still some solubility problems that may affect the bioavailability of the drug. Although the KAE liposome hydrogel showed good homogeneity and stability during the preparation process, it has a high viscosity, which may affect the uniformity of the application and patients’ use and experience in future clinical applications. In the future, we can adjust the composition of the LPs and the preparation process, which can further improve the solubility and stability of the KAE. In addition, the viscosity of hydrogels can be reduced by adding hydrophilic substances to make them easier to apply and use.

## 4. Materials and Methods

### 4.1. Materials

KAE (97%, Shanghai Macklin Biochemical Co., Ltd., Shanghai, China), soy lecithin (Shanghai Jinsong Industry Co., Ltd., Shanghai, China), cholesterols (Shanghai Jinsong Industry Co., Ltd., Shanghai, China), absolute ethanol (AR, Shanghai RichJoint Chemical Reagents Co., Ltd., Shanghai, China), 1,1-diphenyl-2-picrylhydrazyl (DPPH, 98%, Shanghai Yuanye Biology Science and Technology Co., Ltd., Shanghai, China), FeSO_4_·7H_2_O (AR, Sinopharm Chemical Reagent Co., Ltd., Beijing, China), 2-thiobarbituric acid (TBA, 98%, Shanghai Yuanye Biology Science and Technology Co., Ltd., Shanghai, China), trichloroacetic acid (TCA, AR, Tianjin Damao Chemical Reagent Factory, Tianjin, China), carboxymethyl cellulose (CMC, Tianjin Guangfu Institute of Fine Chemical Industry, Tianjin, China), and Carbopol 934 were used.

### 4.2. Animals

Healthy Kunming female mice (20 ± 2 g) were purchased from the Animal Experimental Center of Anhui University of Chinese Medicine (Hefei, China). All the animal experiments were carried out according to the guidelines approved by the Ethics Committee of Anhui University of Chinese Medicine (Hefei, China). The approval number is AHUCM-mouse-2023136 (20231006).

### 4.3. Methods

#### 4.3.1. Network Pharmacology Analysis

In order to better predict the targets of action of KAE and AE, we adopted the following comprehensive strategy: first, a systematic screening of targets related to the disease AE and targets of action of the drug KAE was performed using the Genecards database (https://www.genecards.org/; accessed on 2 January 2024) and the TCMSP database (http://tcmspw.com/tcmsp.php; accessed on 2 January 2024). By cross-referencing these two databases, we successfully identified 121 KAE-AE common targets and drew a Venn diagram (Figure 1A) using a professional data visualization tool to visualize the distribution of these common targets. To gain a deeper understanding of the interactions of these common targets, we further utilized the STRING database (https://string-db.org; accessed on 2 January 2024). We imported the predicted target data obtained from the screening of KAE acting on AE into the STRING database and downloaded the interaction network data of these targets in the tsv format. Subsequently, we imported the obtained tsv format files into Cytoscape 3.8.2 software (a general software platform for analyzing and visualizing complex networks). In Cytoscape, we constructed a protein–protein interaction (PPI) network (Figure 1B) to visualize the interaction relationship between these common targets and provide an important basis for our subsequent analysis. To deeply analyze the relevant pathways of these crossover genes, we utilized the Database for Annotation, Visualization, and Integrated Discovery (david.ncifcrf.gov; accessed on 2 January 2024). By performing pathway analysis on these crossover genes, we obtained information on the biological pathways in which they are involved. Meanwhile, in addition to bioinformatics resources (e.g., bioinformatics.com.cn, accessed on 2 January 2024), we selected core targets to enrich the pathway information further and search for potential targets of KAE for AE. Finally, to verify the biological significance of these potential targets, we performed GO (Gene Ontology) and KEGG (Kyoto Encyclopedia of Genes and Genomes) enrichment analyses. GO enrichment analyses provided information on the biological processes, cellular components, and molecular functions in which these targets were involved, while KEGG enrichment analyses revealed the metabolic pathways and signaling pathways in which they were involved. These results provide important clues for us to understand the molecular mechanism of the KAE treatment for AE and provide a theoretical basis for subsequent experimental validation.

#### 4.3.2. Molecular Docking Analysis

Screening of the key active ingredients in the “active drug–active ingredient cross-target” network and final screening of the core targets for molecular docking validation: Data for the active compounds in KAE were downloaded from the PubChem website in SDF format and exported as small-molecule-compound files, which were converted to pdb format using Open Babel. Data for the core proteins MAPK, JAK, and STAT3 were then downloaded from the UniProt database, and the three-dimensional structures of the proteins were pre-processed using AutodockTools software1.5.7. This step involves removing small-molecule ligands and water molecules from the protein structure and preserving only the 3D structure of the protein receptor. Then, the protein receptor was hydrated, and the charge of the protein receptor was calculated. After these processes were completed, the small-molecule ligand (i.e., the KAE active compound) and the protein receptor were saved as a pdbqt format file, and the grid parameters and the size of the axes of the protein receptor were set using GridBox to find the binding sites of the protein receptor and the small-molecule ligand. VINA software1.2.5 was used to obtain the corresponding binding sites and binding energies. The conformational maps of the molecular docking were visualized and analyzed using PyMOL software3.0.4.

#### 4.3.3. Standard Curve of KAE

First, 0.01 g of KAE was accurately weighed and dissolved in 2 mL of absolute ethanol and 8 mL of 0.2% CMC (at a volumetric ratio of 1:4). From this mother liquor, five solutions, at concentrations from 0.01 to 0.1 mg/mL, were prepared. The absorbances of these solutions at 360 nm were determined using a UV spectrophotometer (1600 UV-Vis, Shanghai Mepeda Instrument Co., Ltd., Shanghai, China) and recorded to establish a standard curve.

#### 4.3.4. Preparation of Kaempferol-Loaded Liposomes

Kaempferol-loaded liposomes (KAE-LPs) were prepared using a thin-film hydration method. First, 0.30 g of lecithin and 0.10 g of cholesterol were accurately weighed into a 250 mL beaker, and 5 mL of anhydrous ethanol was added to dissolve them completely with the aid of ultrasound. The temperature of the rotary evaporator was set at 70 °C, the vacuum pressure was 70 kPa, and the rotational speed was 100 r/min until the anhydrous ethanol was completely evaporated and a thin film was formed on the wall of the round-bottomed flask; then, 10 mL of KAE (2.5 mg/mL) solution was added. The mixture was stirred on a magnetic stirrer to obtain a homogeneous mixture, and the solution was transferred to a 10 mL test tube. Blank LPs (Blank-LPs) were prepared in the same manner but without the addition of the KAE.

#### 4.3.5. Particle Size and Zeta-Potential Measurements of KAE-LPs

The blank-LPs and KAE-LPs were diluted with PBS solution, respectively. The average particle size and zeta-potentials were determined using a nanoparticle size potential analyzer (Malvern Zetasizer Nano ZS90), and the data were recorded. 

#### 4.3.6. Determination of Encapsulation Efficiency of KAE-LPs

The encapsulation efficiency of the KAE in the LPs was determined using the centrifugation technique. First, 1.0 mL of each of the blank-LP and KAE-LP solutions was accurately measured, put into the inner tube of an ultracentrifuge tube, and centrifuged at 10,000 rpm for 30 min. After centrifugation, 0.5 mL of the supernatant was diluted with 3.5 mL of anhydrous ethanol. The absorbance of the free KAE outside the liposomes was measured at 360 nm.

In addition, 1.0 mL of each of the blank-LP and KAE-LP solutions was accurately measured, and 15 mL of absolute ethanol was added for ultrasonic demulsification (5 min). When the bilayer membrane of the liposomes was completely destroyed, the absorbance of the total amount of KAE in the liposomes was measured at 360 nm. The encapsulation efficiency (EE) of the KAE-LPs was calculated using Equation (1) as follows:

EE% = ((A − B)/A) × 100%(1)

where “A” is the total amount of the KAE inside and outside the LPs, and “B” is the amount of free KAE outside the LPs.

#### 4.3.7. Preparation of KAE-Loaded Hydrogel and KAE-LP-Loaded Hydrogel

First, 0.10 g of Carbopol 934 (CBP-934) was accurately weighed and sprinkled on the surface of 10 mL of 2.5 mg/mL KAE solution, and the mixture was placed on a magnetic stirrer, stirred at 37 °C, and allowed to fully swell overnight to obtain the KAE-loaded hydrogel (KAE-GEL). The blank hydrogel (Blank-GEL) was prepared in the same way without KAE. The KAE-LP-loaded hydrogel (KAE-LP-GEL) was prepared following the procedures described in our laboratory’s previous report [30,31]. In brief, KAE-LPs were mixed with 0.10 g of CBP-934, and the mixture was placed on a magnetic stirrer to fully expand. The blank liposome hydrogel (blank-LP-GEL) was prepared in the same manner without KAE.

Measurement of the viscosity of the hydrogel: The viscosity of 5 mL of water was measured using a capillary viscometer. Then, 1 mL of each of the blank-GEL, KAE-GEL, blank-LP-GEL, and KAE-LP-GEL solutions was placed in a test tube, 4 mL of phosphate-buffered saline (PBS, pH 7.4) was added, and the resulting solution was mixed and measured using a capillary viscometer. The time was recorded, and three parallel operations were performed. The viscosity of the hydrogel was calculated according to Equation (2) as follows:

(2) $$\eta_{2}=\frac{\rho_{2}t_{2}}{\rho_{1}t_{1}}\times \eta_{1}$$

where “η” represents the viscosity (mPa·s), “ρ” represents the density (g/mL), “t” represents the time (s), “1” represents the water, and “2” represents the sample.

#### 4.3.8. Establishment of the Model of AE

Preparation of the sensitizing solution: First, 2,4-dinitrochlorobenzene (DNCB) was weighed and dissolved in a mixture of acetone and olive oil (3:1) to make a concentration of 5% DNCB solution. The 5% DNCB solution was diluted 10 times with the mixture of acetone and olive oil to make 0.5% DNCB solution, and this prepared solution was placed in the refrigerator at 4 °C and kept in reserve.

**Figure 9:**
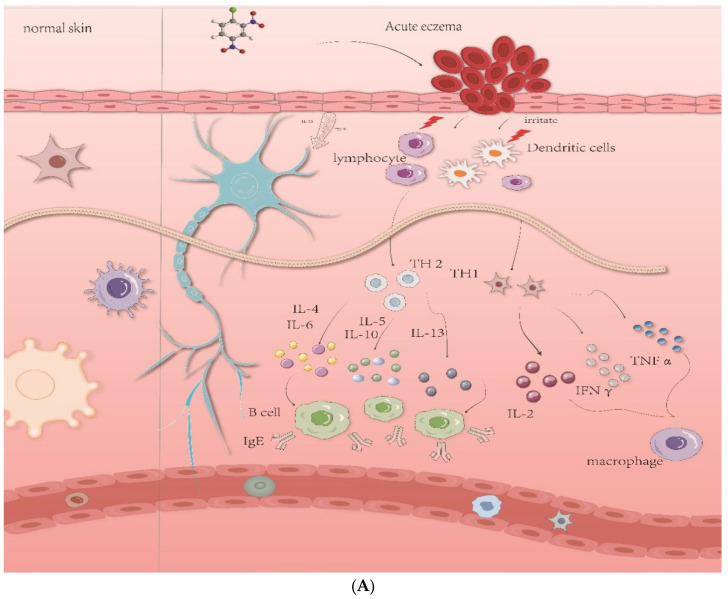

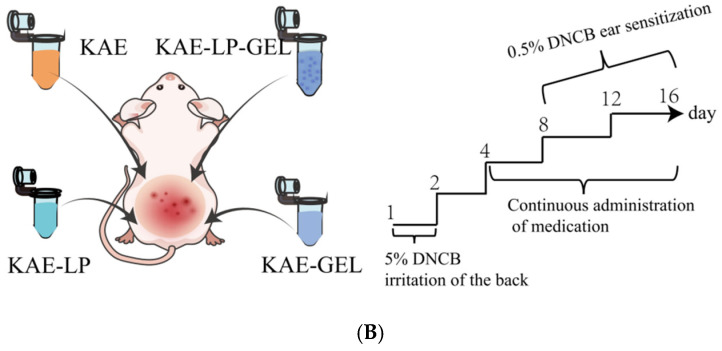
Modeling and experimental advancement in AE. (**A**) Acute response of DNCB-induced eczema mouse model; (**B**) experimental progress of DNCB-induced eczema mouse model and drug therapy.

The AE model was established by referring to the method in [32] with some slight modifications. The mice were kept in the animal room for three days before modeling to adapt to the new environment and the experimental progress for establishing the DNCB-induced eczema mouse model and drug treatment, as shown in Figure 9A. One day before the experiment, mouse hair was removed from the backs of the mice with an electric hairdresser and hair removal cream, and the hair on the back of each mouse was shaved into a block 3 cm wide × 3 cm long. All the groups of mice except the mice in the blank group were sensitized by applying 100 μL of 5% DNCB to the dorsal hair removal site and sensitized on days 1 and 2. After the first induction, the mice were kept normally. After five days, the 0.5% DNCB solution was applied to the skin of the ear for re-sensitization, which was performed every three days and required sensitization three times, and the mice in the blank group were coated with equal amounts of acetone solvent. On day 4, all the mice were treated with PBS, compound dexamethasone acetate cream, KAE, KAE-LP, KAE-GEL, or KAE-LP-GEL, once daily for 12 days of administration, as shown in Figure 9B. If sensitization occurred on the same day, the drug was administered 2 h before the sensitization. Then, 24 h after the end of the last treatment, the mice were anesthetized using an intraperitoneal injection of 20% ethyl carbamate (0.2 mL/20 g), and the right and left ears, organ tissues, and dorsal skin were collected for subsequent testing.

#### 4.3.9. Bodyweight and Skin Thickness Measurements

Each day, before the experiment, the weight of all the mice was measured and recorded, and the skin thickness on the backs of the mice was measured with a Vernier caliper.

#### 4.3.10. Determination of Scratching Times

On the 16th day, after sensitization, the numbers of scratches on the ears and backs of the mice within 20 min were recorded, and the continuous scratch count was once.

##### Scratching Behavior

A mouse raised its paw and scratched it continuously for a long time until the paw returned to the floor, which was recorded as a scratching behavior.

#### 4.3.11. Detection of the Scores of Skin Inflammation

In order to evaluate the scores of skin inflammation after treatments, the signs of the skin lesions on the backs of the mice were observed and recorded visually, and the skin lesions were divided into two categories: erythema and edema/scab. The information of the mouse groups was hidden before scoring, and the severity of the skin lesions in each group of mice was scored according to Table 3, using a six-level scoring method, from 0 to 5. The average skin injury score of the mice in each group is shown in Table 3.

#### 4.3.12. Determination of Ear Swelling

On the seventeenth day, the mice were killed after anesthesia, the left and right ears of the mice were cut off. Ear pieces with the same area were prepared from the left and right ears with a hole punch with a diameter of 6 mm; the weight was quickly measured on an analytical balance, accurate to 0.0001 g, and the data were recorded. The ear-swelling degree and swelling inhibition rate in each group of mice were calculated according to Formula (3) as follows:

(3) $$\mathrm{Inhibition}\;\mathrm{rate}\;\mathrm{of}\;\mathrm{ear}\;\mathrm{swelling}\;(\%)=\frac{weight\;of\;left\;ear\;with\;inflammation-weight\;of\;healthy\;right\;ear}{weight\;of\;healthy\;right\;ear}\times 100\%$$

#### 4.3.13. Determination of Spleen and Thymus Indices

On the seventeenth day, each mouse was weighed, and the mice were killed after anesthesia. The spleen and thymus tissues were dissected and taken out, and the residual blood was absorbed using filter paper and weighed immediately. The spleen and thymus indices in each group of mice were calculated according to the following organ index formula:

(4) $$\mathrm{Organ}\;\mathrm{index}\;(\mathrm{mg}/g)=\frac{Organ\;weight}{Mouse\;body\;weight}\times 100\%$$

#### 4.3.14. Observation of Histopathological Sections

Hematoxylin and eosin (HE) staining was used to detect the back-skin tissue sections of the mice in each group to evaluate the thickness and structure of the back skin of the mice with AE and to evaluate the therapeutic effect of the KAE-LP-loaded hydrogel. On the 17th day, a part of the back skin from each group of mice was cut with scissors and fixed with 4% paraformaldehyde for a paraffin-embedded section.

First, the paraffin slices were dewaxed in water for later use, and the process was as follows: (1) dewaxing in xylene for 15 min; (2) further dewaxing in fresh xylene for 15 min, (3) anhydrous ethanol for 5 min, (4) 90% ethanol for 5 min, (5) 80% ethanol for 5 min, (6) 70% ethanol for 5 min, (7) and distilled water for 5 min. After the last step, the slices were placed on an iron frame for subsequent dyeing steps as follows: (1) hematoxylin for 7 min; (2) tap water was used to flush the excess dye solution; (3) eosin for 10 s; (4) tap water was used to flush the excess dye solution; (5) the xylene was transparent for 3 min. After air drying, the tablets were sealed with neutral gum, and after the gum solidified, they were observed using the platform of an intelligent tissue slice image analysis system.

#### 4.3.15. Determination of MDA Contents in Skin and Tissues In Vitro

##### Drawing of the MDA Standard Curve

First, 0.315 g of 1,1,3,3-tetraethoxypropane was weighed, dissolved, and diluted to 1000 mL to prepare a 100 μg/mL MDA standard solution. Five diluted MDA standard solutions with different MDA concentrations (0.4, 0.8, 1.2, 1.6, and 2 μg/mL) were prepared with distilled water. Then, 3 mL of a mixed solution of TBA and TCA was added, and the resulting mixture was heated in a boiling water bath for 30 min, cooled under running water, and centrifuged. The absorbance of the supernatant was measured at 532 nm, and the standard curve was drawn.

The determination method of the MDA contents in the skin and tissues was just to induce lipid peroxidation without adding Fe^2+^, directly heat the mixture in a water bath, and measure the absorbance value at a wavelength of 532 nm after centrifugation, adjust it to zero with normal saline, and convert it to the MDA content according to the MDA standard curve.

#### 4.3.16. Statistical Methods

All the data were expressed as means ± standard deviations (x ± s); one-way ANOVA was used, and the LSD method was used for two-way comparisons between groups. The test level was α = 0.05, and the difference was considered as statistically significant at *p* < 0.05.

## Figures and Tables

**Table 1:** Physical properties of different formulations.

**Sample**	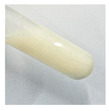	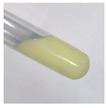	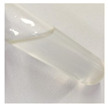	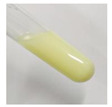	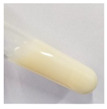	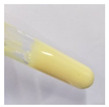
	**Blank-LP**	**KAE-LP**	**Blank-GEL**	**KAE-GEL**	**Blank-LP-GEL**	**KAE-LP-GEL**
Color	milky white	yellow	colorless	bright yellow	milky white	faint yellow
Viscosity (mPa·s)	-	-	2.667	3.238	3.489	3.706

**Table 2:** The ear swelling of the mice in each group.

Group	Ear Symptoms		Left Ear (g)	Right Ear (g)	Swelling Degree (g)	Swelling Inhibition Rate (%)
	Left Ear	Right Ear				
Blank	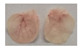		0.0080 ± 0.0014	0.0077 ± 0.0013	0.0003 ± 0.0002	4.78 ± 2.14
Model	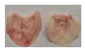		0.0111 ± 0.0005	0.0070 ± 0.0006	0.0041 ± 0.0001	59.16 ± 5.69 ####
Positive	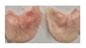		0.0087 ± 0.0012	0.0070 ± 0.0013	0.0017 ± 0.0003	25.02 ± 6.93 ****
KAE	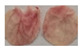		0.0096 ± 0.0008	0.0068 ± 0.0007	0.0029 ± 0.0004	42.66 ± 7.59 *
KAE-LP	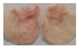		0.0098 ± 0.0012	0.0072 ± 0.0011	0.0026 ± 0.0001	35.96 ± 4.67 **
KAE-GEL	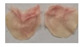		0.0100 ± 0.0007	0.0074 ± 0.0005	0.0020 ± 0.0002	27.67 ± 3.35 **
KAE-LP -GEL	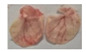		0.0093 ± 0.0006	0.0073 ± 0.0005	0.0020 ± 0.0002	27.86 ± 2.38 ***

Notes: Compared with the blank group, #### *p* < 0.0001; compared with the model group, * *p* < 0.05, ** *p* < 0.01, *** *p* < 0.001, and **** *p* < 0.0001.

**Table 3:** Degrees of skin lesions and scores of mice in each group.

Skin Reaction	Points
Erythema:	
Erythema-free reaction	0
Mild erythema reaction (barely visible)	1
Moderate erythema reaction (visible)	2
Severe erythema reaction	3
Mild eschar formation, purplish red erythema	4
Severe eschar formation, purplish red erythema	5
Edema/Scab:	
No edema reaction	0
Mild edema reaction (barely visible)	1
Moderate edema (pronounced bulge)	2
Severe edema reaction (the skin has a clear bulge, 1 mm)	3
Severe edema (the skin has a bulge of about 1 mm, in the large range)	4
Very severe edema with exudation and scab	5

## Data Availability

The data will be made available on request.
